# The Microbiota and Gut-Related Disorders: Insights from Animal Models

**DOI:** 10.3390/cells9112401

**Published:** 2020-11-02

**Authors:** Layla Kamareddine, Hoda Najjar, Muhammad Umar Sohail, Hadil Abdulkader, Maha Al-Asmakh

**Affiliations:** 1 Department of Biomedical Science, College of Health Sciences, QU Health, Qatar University, P.O. Box 2713 Doha, Qatar; lkamareddine@qu.edu.qa (L.K.); Hnajjar@qu.edu.qa (H.N.); msohail@qu.edu.qa (M.U.S.); ha1305202@qu.edu.qa (H.A.); 2 Biomedical Research Center, QU Health, Qatar University, P.O. Box 2713 Doha, Qatar

**Keywords:** animal models, germ-free, gut microbiota, gut-related disorders, host–defense, pathogen virulence

## Abstract

Over the past decade, the scientific committee has called for broadening our horizons in understanding host–microbe interactions and infectious disease progression. Owing to the fact that the human gut harbors trillions of microbes that exhibit various roles including the production of vitamins, absorption of nutrients, pathogen displacement, and development of the host immune system, particular attention has been given to the use of germ-free (GF) animal models in unraveling the effect of the gut microbiota on the physiology and pathophysiology of the host. In this review, we discuss common methods used to generate GF fruit fly, zebrafish, and mice model systems and highlight the use of these GF model organisms in addressing the role of gut-microbiota in gut-related disorders (metabolic diseases, inflammatory bowel disease, and cancer), and in activating host defense mechanisms and amending pathogenic virulence.

## 1. Introduction

Humans develop a symbiotic relationship with microbes at an early age [1]. This relationship progresses, in turn, into a gradual edification of an elaborate ecosystem known as the human microbiota which contains more than 100 trillion microorganisms that crucially tip the balance towards health or disease [2,3]. Diverse microbial communities are involved in several physiological processes in a host, including immune cell development, fermentation and food digestion, maintenance of metabolic homeostasis, and angiogenesis [4]. As such, considerable attention has been given to understanding the association between the microbiota and a broad range of human pathologies, including metabolic diseases like obesity and diabetes [5,6], inflammatory bowel disease (IBD), and cancer [7,8,9]. The use of pertinent model systems with varying levels of microbial complexity provides a convivial opportunity for a better insight into the effect of host–microbiota interaction on dictating a state of health or disease in a host, an approach that could not be easily implemented in humans and other higher complex organisms alone. Manipulation of the gut microbiota composition of both –vertebrates and invertebrates allows functional and mechanistic studies directed towards determining the causality in disease-associated alterations [10]. The gut microbiota-provoked immune maturation has been presented as a chief alignment in perpetuating gut homeostasis by safeguarding the host from injury [11], detrimental inflammation [12,13], and infections [14]. The role of the commensal microbiota in maintaining gut morphology and homeostasis in both vertebrates and invertebrates has been partly attributed to its involvement in modulating cell composition, epithelial renewal, and tissue structure [15,16,17]. The symbiotic gut microbiota has been also profoundly implicated in various aspects of host physiology including lipid and carbohydrate metabolism [18,19,20,21,22,23,24,25,26,27]. Interestingly, the gut microbiota also exhibits a complex bidirectional relation with non-antibiotic drugs. Although the gut microbiota composition is affected by the uptake of some commonly used drugs [28,29], the commensal microbiota itself influences an individual’s response to drugs by enzymatically altering the drug’s structure, therefore affecting its toxicity, bioactivity, and bioavailability [30]. The gut microbiota can also indirectly impinge a cancer patient’s response to immunotherapy by affecting the patient’s general immune status [31].

Germ-free (GF) model organisms present one of the fundamental means to study the effect of an altered microbial community on the physiological status of a host. Among different model organisms, fruit flies, zebrafish, and mice have been extensively employed in such studies. The advantage of using such model organisms is chalked up to their ease in maintenance, strong genetic coherence, physiological and signaling pathways resemblance to humans, powerful manipulatable genetic systems, and culturable microbiota.

In this review, we shed more light on the use of these model systems to divulge the role of the gut microbiota in activating host immunity and amending pathogenic virulence, and to unravel the role of the gut microbiota in the development and progression of gut-related disorders.

## 2. Provisos of Selecting Animal Models to Study Gut-Related Disorders

Several international laws governing animals′ use in research and testing mandate replacing animal experiments, wherever reasonably feasible, by humane alternatives. To address a scientific question, the primary choice of researchers could be initially directed towards employing non-animal technologies, such as in silico or in vitro approaches [32]. Recently, research is underway to develop computer simulations and gastrointestinal organoids as one approach to further refine the use of animal models [33,34]. The use of animal models to address complex biological problems should not merely be viewed as a technical utility and is therefore generally paralleled with ethical implications considered with risks, animal and public health safety, and interpretability and reproducibility of the results. It is indeed the researcher’s moral duty to honor animals and give due consideration to their capacities for distress when being manipulated. For example, the production and maintenance of GF animal colonies and the induction of metabolic diseases by oral or invasive procedures greatly aggravate animals’ suffering. Therefore, it is extremely desired to follow standard provisions to protect the animals used in such practices from discomfort, misery, trauma, or permanent harm, and to ensure that, where possible, their suffering is kept to a minimum. The use of animals shall only be permissible if the work promises to contribute to a fundamental understanding of important biological concepts or to the advancement of knowledge that can reasonably be expected to benefit both humans and animals. As such, the 3Rs (Replacement, Reduction, and Refinement) set by Russell and Burch [35] shall be strictly followed to minimize animal suffering and usage.

If the scientific objective raised can be only accomplished by using an animal model, then a comprehensive assessment of the pros and cons should be conducted to identify and appraise the most suitable animal model. Turner et al. [36], for instance, reviewed the human gut microbiota model’s application in rats, mice, guinea pigs, minipigs, conventional swine, dogs, and non-human primates and concluded that murine models bear the closest microbiota resemblance to humans. In this context as well, fecal microbial transplant studies of humanized germ-free murine models have shown that rats have more similar microbiota with humans than mice [37]. One reasoning behind that could be attributed to the belief that rats have more human baseline microbiota than mice, enabling the inoculated bacteria to be more stable in expression and development [38]. The lack of genetic variants in rats might also restrict their utility in studying specific models of diseases [39]. Therefore, genetic background, baseline microbiota resemblance, and phenotypic expression of the diseases studied is generally considered while selecting the most suitable animal model. The decision-making process regarding which animal model is most appropriate should also emphasize which species and modelsare most likely to provide the most translatable and valid data and results. Some of the most common justifications provided for the selection of animal models such as ease of maintenance and low cost might not be always perceived as adequate, and therefore additional thought should be given when deciding on the choice of the animal model with the most translatable outcomes.

## 3. Fruit Fly

*Drosophila*, commonly known as the fruit fly, is one of the most powerful model organisms used in various research areas. Fruit flies are easily cultured and maintained in a laboratory, have a relatively short life cycle, and yield many offspring in a short period of time [40]. *Drosophila*’s sequenced genome has additionally allowed its genetic manipulation and the discovery of a number of genes whose homologs cause diseases in humans [41]. In recent years, the use of *Drosophila* has been most commonly implicated in genetics, drug screening and toxicity, immunology, and metabolism related studies [42]. The fruit fly has also gained particular attention for its use in understanding gut-associated disorders due to the structural and functional similarities between a fly and a vertebrate gut [43,44]. Into the bargain, the adjacent concordance between the fly gut-brain axis advocated the powerful contribution of the gut microbiota to the fly’s behavior and its physiological processes [45]. Dysbiosis of the fly gut has been associated with cancer [46], IBD occurrence [47], lifespan alteration, and overall developmental progression [42]. Several studies have also employed *Drosophila* as the model of choice to understand the host defensive mechanisms against different intestinal pathogens like *Vibrio cholerae* [48], *Serratia marcescens* [49], *Pseudomonas aeruginosa* [50], and *Enterococcus faecalis* [51]. Many of these studies also correlated metabolic adaptations with the host’s response to infection [52], and depicted that alterations in the intestinal microbiota can have a profound impact on the host’s normal physiological processes and may contribute to disease progression [53].

### 3.1. The Fruit Fly Gut and Microbiota

**Figure 1:**
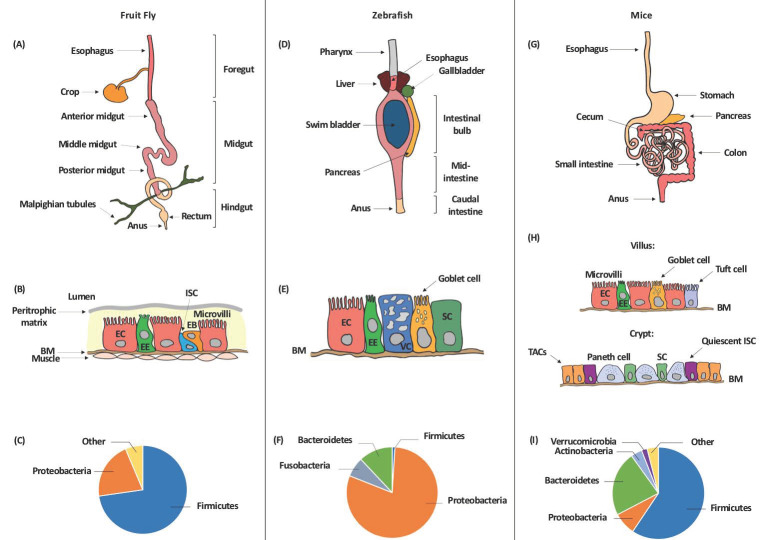
The gut structure and normal flora of different model organisms including (**A**–**C**) the fruit fly, (**D**–**F**) zebrafish, and (**G**–**I**) mouse. (**A**,**D**,**G**) Anatomy of the gut; (**B**,**E**,**H**) cell types present, including enterocytes (EC), enteroendocrine cells (EE), intestinal stem cells (ISC), enteroblasts (EB), goblet cells, stem cells (SC), vacuolated cells (VC) and, in the mouse, chemosensory tuft cells in the villi and paneth cells, transit amplifying cells (TAC), and quiescent ISC in the crypts; (**C**,**F**,**I**) composition of the major phyla according to [59,60,61]. BM = basement membrane.

The *Drosophila* gut consists of three main regions: the foregut, the midgut, and the hindgut. The foregut includes the oral cavity, esophagus, and crop in adult flies where food is ingested and initially processed by the enzymes released. Further digestion and assimilation of the food by enzymes including carbohydrases, proteases, and lipases occurs in the midgut. The hindgut regulates the absorption of water, ions, and other nutrients, specifically those released from the Malpighian tubules, a structure equivalent to the kidneys in vertebrates [54] (Figure 1A). The epithelial cell lining of the fly gut is composed of four cell types including enterocytes (ECs), enteroendocrine cells (EEs), intestinal stem cells (ISCs), and immature progenitor enteroblasts (EBs), which mainly differentiate as ECs or possibly as EEs [55,56,57] (Figure 1B). The microbiota of a fly is separated from its epithelial cells by a chitinous layer, instead of a mucus one, its hindgut is not a fermentation cavity similar to a mammalian colon, and its gut is well-thought-out as an unsuitable milieu for obligate anaerobic. As such, the *Drosophila* gut may not be colonized with most gut microbes needed to develop a humanized microbiota model. Several standing points based on this could argue that a humanized *Drosophila*-microbiota model might not be very informative in the interpolation of complex metabolic diseases, and is instead more relevant to basic screening studies of host–pathogen interactions [54,58].

Studies on the composition of the gut microbiota have primarily focused on the identification of bacterial species and operational taxonomic units (OTUs) through laboratory cultures and 16S rRNA gene amplicon sequencing [53]. The diversity of the microbial community in a *Drosophila* gut is relatively less as compared to that of mammals, which is not a miniaturized version of the human gut microbiota. Although a sole reason behind such a simplicity in fly’s microbial diversity has not been delineated; yet, it is thought that a higher microbial diversity may be attributable to the type of food [62] and to the presence of an adaptive immune system in mammals but not in *Drosophila* [63]. Several factors including changes in gut oxygen tension or pH, host factors including genotype, development and age, physiology and immune response, geographical locations, disturbance, and environmental encounters might also explain the low-diversity of the microbiota community in the *Drosophila* gut [59,64,65,66,67,68]. The microbiota of laboratory-reared flies fed complex polysaccharides diet such as cornmeal and soy flour, for instance, have high abundance of *Lactobacillus* (Firmicutes of the order Lactobacillales), while those fed sugar-rich diets have *Acetobacteraceae* (α-Proteobacteria), specifically *Acetobacter* and *Gluconobacter* species, as the dominant bacterial group [54,68]. Some fly cultures are also highly abundant with γ-Proteobacteria or *Enterococcus* to the point of eliminating or nearly eliminating *Acetobacteraceae* and *Lactobacillus*. Several taxonomic differences in the microbiota also exist between wild *Drosophila* populations and those reared in a laboratory. Although *Acetobacteraceae*, *Lactobacillales*, and γ-Proteobacteria also dominate the gut microbiota of natural *Drosophila* populations, the gut microbiota of wild flies is much more diverse [69,70,71,72]. Additionally, the number of *Lactobacillus* tends to be low or undetected in natural *Drosophila* populations, with only *Leuconostoc*, *Enterococcus*, and *Weissella* representing *Lactobacillales* being frequently abundant [73]. Though poorly studied, yeasts, mainly *Hanseniaspora*, *Pichia*, and *Candida*, have been also detected in the gut of both wild-caught and laboratory-reared *Drosophila*, particularly those fed rotting fruits [74,75,76] (Figure 1C).

### 3.2. Generation of GF Fruit Flies

The generation of GF (axenic) and gnotobiotic model organisms, including flies, has greatly contributed to our understanding of the role of the gut microbiota in maintaining a normal physiological status of a host [77]. Generating GF flies can be achieved in different ways including egg dechorionation (removal of the eggshell layer), rearing flies on a broad-spectrum antibiotic cocktail-containing diet or placing flies on an axenic diet. In egg dechorionation, the first step involves egg laying of cage flies on an agar plate. Eggs are then collected with a sterile paintbrush and embryos are subsequently washed with hypochlorite solution, followed by water. The time of hypochlorite treatment varies between protocols and some dechorionation procedures also include ethanol washes post hypochlorite rinse [78,79,80,81,82]. Embryos are then carefully transferred with a paintbrush to axenic media. Since removing endosymbionts like *Wolbachia* and *Spiroplasma* by bleaching remains challenging, the tetracycline antibiotic is usually added to the axenic media to aid in this removal [82]. Flies serially transferred to axenic media can remain GF for multiple generations [80,82].

**Figure 2:**
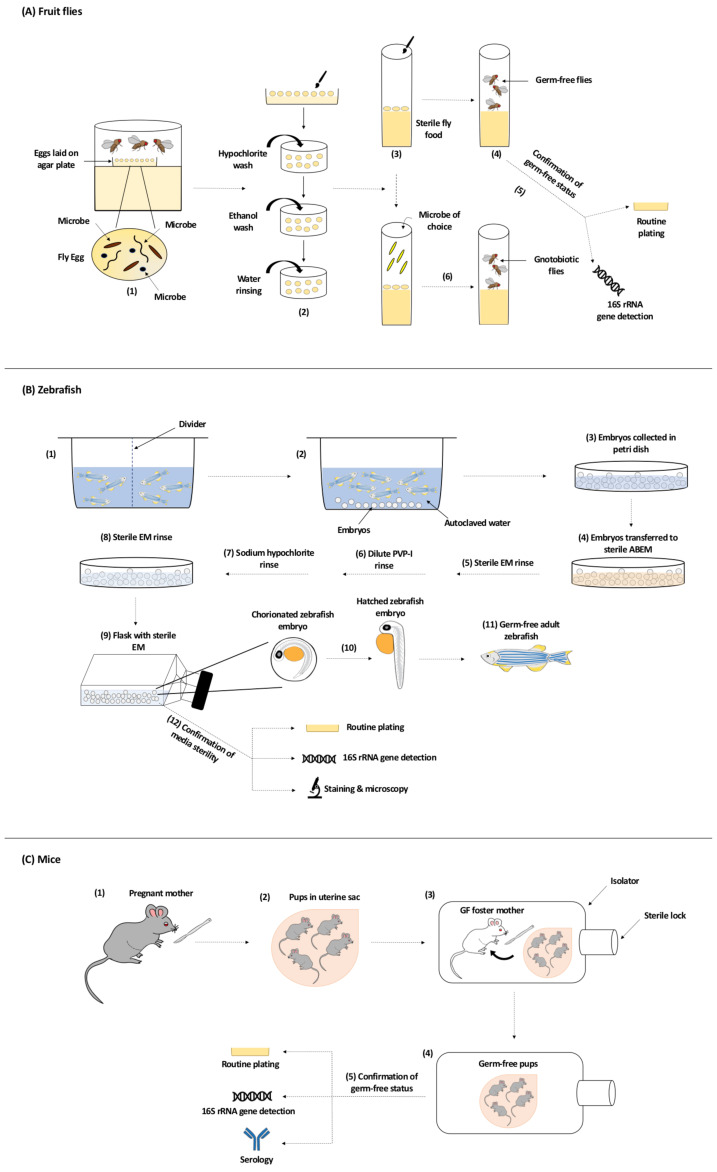
Generation of Germ-Free Model Organisms. (**A**) Generation of Germ-Free Fruit Flies. Flies trapped in a cage lay their eggs on the agar plate. The surface of the laid eggs harbors microorganisms from maternal fecal deposits (1). Using a sterile paint brush, eggs are collected from the agar plate before larval formation, dechorionated with hypochlorite solution, and rinsed with water. An ethanol wash between the hypochlorite dechorionation step and the water rinsing step could be done (2). Dechorionated eggs are transferred to sterile fly food (3) and germ-free flies are generated (4). The most common methods used to confirm the germ-free status of flies include routine culturing on selective media permissive to the growth of gut bacteria and 16S rRNA gene detection via PCR amplification and sequencing (5). To generate gnotobiotic flies, microbe of choice is added to the sterile fly food vial containing dechorionated eggs (6). (**B**) Generation of Germ-Free Zebrafish. Adult zebrafish are set up in breeding cages overnight with dividers to prevent breeding and spawning (1). The next day, adults are transferred to tanks with autoclaved water and dividers are removed to allow breeding and spawning for less than an hour. Embryos are deposited in the tanks (2) and are then collected into petri dishes and rinsed thoroughly to remove any debris (3). In a biosafety cabinet, zebrafish embryos are transferred to petri dishes with sterile antibiotic embryo media (ABEM) (4) and undergo a sterile embryo media (EM) rinse (3 times) (5), followed by a dilute polyvinylpyrrolidone-iodine (PVP-I) rinse (6), and finally a sodium hypochlorite rinse (7). Embryos are then rinsed with sterile EM (3 times) again (8) before being transferred into sterile tissue culture flasks containing sterile EM where they will be raised (9). If hatching (10) has not occurred within 3 days post-fertilization, gentle flicking of the flask can mechanically dechorionated the embryos. Embryos eventually develop into germ-free adult zebrafish (11). Commonly used methods to test the sterility of the zebrafish media are routine culturing on various selective media under aerobic and anaerobic conditions, 16S rRNA gene detection via PCR amplification and sequencing, and staining and microscopy procedures such as fluorescence in situ hybridization and Gram stain (12). (**C**) Generation of Germ-Free Mice. Pups are removed from their mother by Caesarean section (1) and while still in the uterine sac (2) transferred to a germ-free foster mother (also raised germ-free) in an isolator (3). The first generation is not used for experiments, since the mother was not germ-free and may have transmitted microbes transplacentally to the fetus. Further generations of pups are delivered and cleaned inside this isolator for experimental use (4). The germ-free status of the animals is monitored by routine plating of stool material, detection of 16S rRNA by PCR amplification and sequencing, and serological techniques (5).

Gnotobiotic or conventionalized flies can be produced following the same procedure of axenic flies; yet, after the hypochlorite wash and aseptic transfer of dechorionated eggs to sterile food, microbes of choice are added to the vials housing GF flies. Upon larval and adult feeding on microbe(s) the association between the fly and the microbe(s) gets established and microbes readily colonize the fly gut [54,80] (Figure 2A). Supplementing fly food with a broad spectrum antibiotic such as streptomycin or tetracycline or a combination of antibiotics is another approach to alter the gut microbial community and eliminate the microbiota [83,84,85]. Besides egg dechorionation and antibiotic containing diet, placing flies on axenic food is also another approach used to suppress or eliminate the gut microbiota. The most common methods used to verify a GF status of a fly include routine culture on selective media like de Man, Rogosa, and Sharpe (MRS) [86], and PCR amplification and sequencing of 16S rRNA gene regions [68,86].

### 3.3. The Role of the Gut Microbiota in Fruit Fly–Pathogen Interactions

A fruit fly serves as an important model organism in host–pathogen interaction studies [87]. Although details on the relationship between the microbiota and pathogen virulence are still emerging, studies clearly designate a role of commensals in host resistance to infection. Blum et al. has shown that flies with an intact microbiota are less susceptible than GF flies to *Serratia marcescens* and *Pseudomonas aeruginosa* infections. Captivatingly, augmenting the normal microbiota with higher populations of the *Lactobacillus plantarum* commensal further protects the fly from infection [59]. In response to pathogenic infection, *Drosophila* induces several immune responses, one of which is the production of reactive oxygen species (ROS) to fight off invading pathogens [55,88]. Besides its role against pathogens, the production of ROS also contributes to wound healing, tissue repair, and haematopoiesis by acting as signaling modulators or second messengers [89,90,91]. ROS production is induced by the two NADPH enzymes: dual oxidase (Duox) and NADPH oxidase (Nox) [92]. Studies in *Drosophila melanogaster* have shown that both commensals and pathogenic microorganisms induce ROS production by activating Nox and Duox, respectively [88,93,94]. Flies lacking Duox activity are less resistant to enteric pathogenic infections and succumb to death faster when ingesting microbe-contaminated food [88]. Similarly, ingesting commensals by *Drosophila* and mice, particularly *Lactobacillus* spp., induce Nox1-dependent ROS production and consequently promote intestinal stem cell proliferation [94]. *Lactobacillus platarum* has been presented as a strong activator of the ROS-sensitive CncC/Nrf2 signaling pathway within enterocytes [95]. CncC-dependent gut expression of the cytokine *upd2*, the gene product involved in regulating intestinal homeostasis via JAK/STAT signaling [96], is up-regulated in the midgut tissue of *Lactobacillus plantarum*-fed larvae. Consistently, reduction of Upd2 revokes the ability of *Lactobacillus plantarum* to induce epithelial proliferation in the *Drosophila* midgut [95]. This is verily supported by the notion that indigenous bacteria condition the basal level of epithelium renewal by stimulating intestinal stem cell division, plausibly through elevating JAK–STAT and JNK activity. The JAK–STAT ligand *upd3* is not expressed in the gut of GF flies [16,97].

ROS and AMPs production are thought to complement each other during host defense, where AMPs production contributes to the control of ROS-resistant bacteria [98]. Systemic control of AMP production is mainly nuclear factor-κB (NF-κB) dependent, unlike the regulation of AMPs production in the gut, which is more complex [92]. The gut commensal microbiota plays a role in eliciting IMD-Relish gut immune response. The nuclear translocation of Relish detected in the intestinal cells of conventionally raised flies (with and without gut *Erwinia carotovora carotovora-15* infection) is almost entirely eliminated in GF, antibiotic-treated, and IMD pathway mutant flies. Moreover, the expression levels of, PGRP-SC and PGRP-LB, is also higher in the gut of conventionally reared wild-type flies as compared to antibiotic-treated and IMD pathway mutant flies [81]. Interestingly, the short chain fatty acid acetate produced by the gut microbiota activates the IMD NF-κB signaling pathway in enteroendocrine cells. The absence of the intestinal microbiota, and therefore the production of dietary acetate, hinders the nuclear translocation of Relish in the fly gut, mimicking the phenotype of *rel^E20^* mutant flies. Down this route, acetate-induced nuclear localization of Relish elevates the expression of several IMD pathway-regulated AMPs including *Diptericin*, *Attacin*, *Cecropin*, and *Drososin* [99]. It is worth noting here that besides its protective effect, the intestinal microbiota may indirectly contribute to the virulence of certain enteric pathogens. The consumption of microbiota produced acetate by *V. cholera*, for instance, affords the pathogen a growth advantage, disrupts metabolic homeostasis, and promotes intestinal steatosis in the infected host [100], a phenotype similar to that seen in GF flies and reversed by acetate supplementation [99,100].

Upon gastrointestinal tract fungal infection, the commensal microbiota aids in host survival plausibly by reducing fungal colonization. This interference in pathogen colonization could be mainly attributed to the adjacent concordance between resident microbiota and host immunity, a reasoning tailored to the observation that *Candida albicans* infection of GF larvae compromises the life span of the cytokine Spätzle or IMD mutant larvae [95]. Although Toll signaling appears to be uninvolved in gut immunity and is rather confined to hemocyte and fat body during systemic infection [101], it has been recently shown that the interplay between microbiota-derived peptidoglycan (translocated from gut lumen into systemic circulation) and constitutive Toll pathway activation in *Klf15* mutants lacking nephrocytes-cells and renal filtration of microbiota-derived peptidoglycan promotes resistance to infection [102]. Besides its contribution to bacterial and fungal intestinal immunity, the microbiota also takes part in priming antiviral gut immunity. To activate antiviral ERK signaling in the intestinal epithelium, two signal inputs are required, one of which is dependent on priming the NF-kB-dependent induction of the secreted factor Pvf2 upon microbiota (particularly *Acetobacter pomorum*) peptidoglycan recognition. This however is followed by a second virus-initiated Cdk9 kinase-dependent signaling required for Pvf2 production and intestinal ERK response [103]. In tsetse flies (*Glossina* spp.), the obligate mutualist *Wigglesworthia* promotes the up-regulation of *odorant binding protein* (obp) *six* expression in the gut of intrauterine tsetse larvae. Such an up-regulation is necessary and sufficient to induce the systemic expression of the hematopoietic RUNX transcription factor *lozenge* and promote the subsequent production of crystal cells involved in the melanotic immune reaction. Enthralling, the indigenous microbiota of *Drosophila* larvae regulates an orthologous hematopoietic pathway. The expression levels of *obp28a* (*Drosophila*’s orthologue of tsetse *obp6*) and *lozenge* is higher in conventionally reared larvae as compared to axenic ones. Conventionally reared larvae also manifest more cuticular sessile crystal cells (a class of hemocytes) and prophenoloxidase, the zymogen form of phenoloxidase, the rate limiting enzyme in the melanotic immune response [104]. Producing hemocyte-free larvae by depleting plasmatocytes and crystal cells causes a shift in immune effector pathways designated by massive lamellocyte differentiation, Toll induction, and IMD repression. This, in turn, drives to a pro-inflammatory state characterized by the formation of melanotic nodules in the hemolymph and to substantial developmental defects. Interestingly, such phenotypes are microbiota-mediated, appear to be modulated by nitric oxide levels, and could be ameliorated by antibiotic administration and change of food source [105,106].

### 3.4. The Role of the Fruit Fly Gut Microbiota in Gut-Related Disorders

An omnipresent facet of all living beings with an open digestive tract is the commensal microbiota colonization of their gastrointestinal swathe [27]. The gut microbiota community flourishes on nutrients from the host’s diet and gut secretions and is sculptured by the gut environs, penchant for certain types of food, and dietary patterns of the host [27,107]. This indigenous microbial community, in turn, takes part in host growth, development, immune regulation, and maintenance of metabolic balance in various ways including metabolites production, modulation of hormonal signals, secretion of essential nutrients, and alteration of nutrient availability [23,27,92,99,108,109]. In *Drosophila melanogaster*, the gut microorganisms (particularly *Acetobacter pomorum* and *Lactobacillus plantarum*) participate in growth and development through insulin signaling [23,24,92,110], a process that is altered by gut microbiota dysbiosis. Interestingly, such a dysbiosis could translate into metabolic disorders in flies, a phenotype similar to the one reported in humans [111]. GF flies exhibit prolonged development time, disrupted insulin signaling and lipid metabolism, a status that could be reversed by the supplementation of the microbial metabolite acetate [24,99,100] or by the generation of gnotobiotic flies [22] colonized with different bacterial taxa. Compellingly, the bacterial taxon needed to recapitulate glucose elevation differs from that needed to maintain normal development rates and triglyceride levels. While the colonization of any one of the five *Acetobacter* and *Lactobacillus* species (*A. pomorum*, *A. tropicalis*, *L. brevis*, *L. fructivorans*, and *L. plantarum*) restores glucose levels to normal, the development rates and triglyceride content in monocolonized flies differ according to the taxon present, where *Acetobacter* species braces the largest reductions, unlike the majority of the *Lactobacillus* species that have no effect. Only flies with both *Acetobacter* and *Lactobacillus* manage to restore triglyceride content to the level detected in conventional flies [22].

By the same token, the microbiota influences the gut morphology by impacting epithelial renewal rate, epithelial cell type composition, and cellular spacing [15]. This contribution of the gut microbiota to the positive regulation of midgut epithelium renewal happens via stem cell proliferation [15,16,24]. Indeed, the induction of intestinal stem cell proliferation and differentiation during steady-state conditions (by microbiota) or to repair epithelium infectious damage (by pathogens) promotes homeostatic responses [16,24,112,113]. Flies lacking such a compensatory proliferation behavior and therefore an active tissue repair process succumb to infection faster. Interestingly, microbes are also involved in skewing cell lineage in the gut. The absence of the gut microbiota results in a decrease in enteroblasts and an increase in enteroendocrine cells in a GF fly gut [15,16,24].

In point of fact, such an alignment between the gut microbiota and epithelial cell proliferation explains listing the microbiota as one aetiological factor for colorectal cancer development [114]. As much as the gut microbiota facilitates several aspects of health and development, a dysbiotic microbiota committee can promote hyperplasia and inflammation [115,116,117,118,119]. In humans for example, *Helicobacter pylori*, a resident bacterium of the stomach, induces gastritis and elevates the risk of developing gastric cancer. *Helicobacter pylori* infections are also associated with alterations in the gastric and colonic microbiota [120]; yet, the contribution of *Helicobacter pylori*-induced dysbiosis to tumor development has not been established. Previous studies have highlighted a role of *Helicobacter pylori* cytotoxin-associated gene A (CagA), a potent virulence factor that modulates several host signaling pathways, including the Ras/ERK MAPK pathway, in disrupting receptor tyrosine kinase signaling and promoting cell proliferation [121]. Besides, CagA was shown to promote pro-inflammatory cytokines expression and alter the host response to infection by inducing inflammatory processes via NF-κB signaling [122]. Interestingly, a recent related study in *Drosophila* delineated the involvement of the gut microbiota in *Helicobacter pylori*-induced tumor development by showing that CagA promotes microbial dysbiosis, contributing to unrestrained epithelial cell proliferation in the fly gut [123].

Several lines of evidence ascribe the disruption of intestinal NF-κB signaling and AMP production to the etiology and pathology of IBD development [124,125,126]. Strikingly, a study by Ryu et al., shows that a defective regulation of AMP levels in flies lacking the developmental master control gene, *Caudal*, exerts a selection pressure that favors the dominance of the pathogenic commensal, *Gluconobacter* sp. strain EW707, promoting gut pathology [81]. This inception of a disease-causing commensal organism in an immune-defective genotype setting advocates presenting a gut microorganism as a plausible cause of chronic inflammatory disorders.

## 4. Zebrafish

Zebrafish (*Danio rerio*) is another key animal model organism whose use in host–microbe interaction studies is being rapidly adopted. This comparatively simple vertebrate organism has several advantages over higher mammals including its rapid growth and high fertility rates, small size, and the economic costs needed for its rearing and maintenance in a laboratory setting. The growing interest of employing zebrafish as the model organism of choice in research is also tailored to its comprehensive genetic analysis, delayed maturation of adaptive immunity enabling innate immunity focused studies, and optical transparency at embryonic and larval stages permitting high-resolution in vivo imaging of developing gut and commensal microbiota [36,127,128,129,130,131].

Like in other animal models, the translation of findings from zebrafish to higher organisms including humans poses several limitations due to many factors, one of which is that zebrafish acquire their commensal microbiota from their aquatic habitats only, rather than from atmospheric and terrestrial sources. Moreover, they are poikilothermic animals that are essentially maintained at 28 °C, limiting their colonization by several microbes detected in homeothermic hosts. Zebrafish also lack mammary glands and lungs which prevents modeling of host–microbiota interactions involving these organs [132]. Nonetheless, and despite all of these limitations, zebrafish still serves as a powerful model to explore interactions between animal hosts and microbiota in states of health and disease.

### 4.1. The Zebrafish Gut and Microbiota

Although the digestive tract of zebrafish lacks a stomach, it resembles that of mammals in many other aspects, including the presence of a liver, gall bladder, pancreas, and intestine with proximal-distal functional specialization. An adult zebrafish intestine consists of one long tube divided into three morphologically distinct sections: the anterior intestine (intestinal bulb), mid-intestine, and caudal intestine (Figure 1D). The anterior segment connects with the esophagus, where the digestion and absorption of nutrients occurs. As in mammals, this absorption of nutrients diminishes gradually in the distal gut, and transportation of essential ions, reabsorption of water, and fermentation occurs only in the middle and posterior segments [133]. The wall of the zebrafish gut is covered by an epithelium containing a single layer of mucus-producing goblet cells, absorptive enterocytes, enteroendocrine cells, and M-like vacuolated cells (Figure 1E), although the submucosal glands, Peyer’s patches, and crypts of lieberkuhn present in mammals are absent in these fish [131,133]. Although paneth cells are also not present in the gut epithelium of zebrafish [134], several β-defensins are expressed at high levels in this tissue [135]. At the molecular level, transcriptomics and gene expression profiling have revealed considerable functional similarity between the digestive tracts of zebrafish, mice, and humans [136,137].

Colonization of the zebrafish gut by bacteria, many of which are detected in the mammalian gut [138], appears to begin at around three days post fertilization (dpf), when the larvae hatch from the axenic environment within their protective chorion [133]. Yet, vertical transmission of the microbiota during oviposition has been also proposed [139]. The core microbiota in zebrafish undergoes specific changes with time and much like *Drosophila*, is dominated by Proteobacteria (76–82%) (Figure 1F). This reflects a considerable difference between its gut microbiota and that of humans and rodents, where Firmicutes and Bacteroidetes represent the dominant phyla [140].

Qualitatively, six of the eleven bacterial phyla found in zebrafish are detected in the gut microbiota of mice and five of these are also shared by the adult human microbiota [138]. However, at higher phylogenetic resolution, the members of these shared phyla are quite different between zebrafish and mammals. When murine microbes (dominated by Firmicutes and Bacteroidetes) get transplanted into GF zebrafish, the small number of Proteobacteria among the bacteria in the mouse intestine grows disproportionately. When GF mice are colonized with the zebrafish microbiota; however, the small numbers of Bacteroidetes and Firmicutes present become dominant. This clearly indicates that the microbiota of these guts is subject to specific selective pressure [138], presumably due to differences in diet, stress, and habitat (which may also vary considerably between different aquaculture facilities in which zebrafish are kept and raised) [127,140,141,142,143]. On the other hand, the microbiota of zebrafish and mammals respond in a similar fashion to a variety of stimuli, including toxins or high dietary levels of fat [144,145]. Despite these differences, much can be learned from studying the zebrafish microbiota.

### 4.2. Generation of GF Zebrafish

It is relatively easy and inexpensive to maintain zebrafish under GF conditions or colonize these animals with specific bacterial species or complex communities (conventionalized). Human commensals including *Bacillus*, *Escherichia*, *Enterococcus*, *Prevotella*, and *Roseburia*, were shown to be able to successfully colonize a GF zebrafish larval gut, providing “humanized” larvae for investigation [138,146]. Furthermore, the zebrafish gut can be colonized by several probiotic bacterial species, including *Bifidobacterium* and *Lactobacilli* [147,148,149].

Zebrafish embryos are generally obtained through natural breeding, or by in vitro fertilization of gametes collected by laparotomy or abdominal squeezing. Laparotomy usually involves minimal exposure of the gametes to the intestinal contents, unlike in squeezing where gametes are subject to transient exposure. To obtain GF embryos using both approaches, in vitro fertilization in a sterile environment is required. Alternatively, the higher number of fertilized embryos obtained through natural breeding can be cleaned, washed, and maintained under GF conditions using well-established procedures. In brief, adult zebrafish breed in a clean cage filled with freshly autoclaved water, and the formed embryos are then transferred to petri dishes containing sterile antibiotic embryo medium (ABEM). After removal of infertile embryos, the viable ones are further incubated for 4–6 h, rinsed thoroughly with ABEM to remove visible debris, and subjected to a series of baths in iodine, antibiotic, and bleach (with intermittent washing in sterile ABEM) to sterilize the surface of the chorion [150]. Subsequently, the embryos are maintained in sterile ABEM, half of which is replaced daily to provide fresh nutrients and eliminate waste, at 28 °C and 14/10 h light/dark cycle (Figure 2B). Axenic larvae eventually develop into adult zebrafish in culture flasks or in a GF isolator fitted with HEPA-filters under positive pressure. Fish are generally maintained in sterile beakers filled with gnotobiotic zebrafish media (GZM; made with 1 L distil water, 7.5 mL sea-salt mixture stock solution, and 1.25 mL neutral pH buffer). Sterile food, water, and other supplies are also placed inside the isolator. Procedures are performed inside the isolator using attached gloves with minimum exposure to the external environment [151]. Otherwise, GF zebrafish can be also maintained in GZM filled in sterile tissue culture flasks inside an air incubator. Flasks are moved into a biosafety cabin to perform any procedure, adapting sterile techniques [152]. By comparing both methods, the use of GF isolator is considered more robust and rigorous, while the tissue culture flask approach is easier to manipulate with many different microbial conditions over a short time course.

### 4.3. The Role of the Gut Microbiota in Zebrafish–Pathogen Interactions

Studies on gnotobiotic animals have revealed that commensal microbes enhance the mass of gut associated lymphoid tissue (GALT), train the host’s immune system, improve the integrity of the intestinal barrier, and modulate the enteric nervous system [129,130]. However, less is known about the effect of microbes on the general physiology of their host. In zebrafish, the growth of the gut wall and expression of genes related to various populations of lymphocytes caused by inoculation of GF zebrafish with conventionalized microbiota represent evolutionarily conserved responses [153].

Zebrafish have innate and adaptive immune systems that resemble those of humans. Innate immunity develops quickly, with macrophage-like cells appearing at approximately 25 h post-fertilization (hpf) [154]. Soon after hatching, the presence of commensal microbes is recognized and promotes immune priming and infiltration of immune cells into the gut. A zebrafish adaptive immune system takes longer to mature; however, providing a unique opportunity to examine initial reactions to intestinal infection [155].

Several studies have used different pathogens such as Salmonella Typhimurium, Shigella flexneri, Vibrio anguillarum, Staphylococcus aureus, Pseudomonas aeruginosa, Mycobacterium marinum, Escherichia coli, and Bacillus subtilis to infect the gut of GF zebrafish [156]. Infection with E. coli, for example, was shown to upregulate the expression of genes related to innate immunity, the proliferation of intestinal cells, and metabolism of nutrients. B. subtilis infection, in turn, enhanced the expression of genes related to stress responses and innate immunity. Interestingly, a dual infection of both bacterial species triggers macrophages and neutrophils-dependent protection against these infections 22 to 36 hpf. Similarly, during the early stages of infection of GF zebrafish with Vibrio anguillarum, the expression of genes encoding interleukin 1 beta (IL-1β), toll-like receptor 4 (TLR4), NF-κB, and transferrin (TRF) decreased significantly [157]. Such findings illustrate the advantages of infecting zebrafish with one specific pathogen at a time. Galindo-Villegas et al. [158] observed that inoculation of GF zebrafish larvae with commensal microbes activates neutrophils and elevates the expression of several pro-inflammatory cytokines via TLR/MyD88 signaling. As in humans that develop typhoid fever, inoculation of zebrafish with S. Typhimurium activates the inflammasomes in neutrophils [159]. Neutrophils recruited through chemotaxis engulf the pathogen, activate cytosolic phospholipase A2, and release prostaglandins. The innate immune system of zebrafish senses commensal microbes by detecting microbe-associated molecular patterns (MAMPs), which bind primarily to toll-like (TLRs) and nucleotide oligomerization domain (NOD)-like receptors (NLRs) [160].

In addition to these cellular immune responses, microbial colonization of zebrafish also activates intestinal alkaline phosphatase (IAP) at the brush border of the intestinal lumen. This enzyme plays an essential role in maintaining gut homeostasis and mucosal defenses, mitigating inflammatory disorders mediated by lipopolysaccharide via dephosphorylating and thus detoxifying thisendotoxin. Moreover, commensal microbiota prevent inflammation through Myd88 and TNRF, which helps in maintaining normal levels of neutrophils in the gut [161].

### 4.4. The Role of the Zebrafish Gut Microbiota in Gut-Related Disorders

The optical clarity of zebrafish during their early development allows fluorescent dyes to be used in monitoring disease-associated changes. In addition, genetic approaches, such as mutations and knockdowns, have been applied to zebrafish in attempt to identify changes associated with human maladies. Several studies have presented zebrafish as a model of choice for studying diabetes, obesity, atherosclerosis, and fatty liver disease [162]. Furthermore, these animals have emerged as a pre-eminent model for studying gastrointestinal diseases and entero-neuroendocrine complications, such as IBD, colitis, Hirschsprung disease, autism spectrum disorder, and chronic intestinal pseudo-obstruction [163,164].

Induction of intestinal inflammation by administering oxazolone to adult zebrafish, for instance, results in IBD [165]. Similarly, trinitrobenzene sulfonic acid impairs intestinal homeostasis in zebrafish larvae, thereby inducing an inflammatory condition resembling human IBD [166]. Fluorescent imaging of zebrafish larvae treated with trinitrobenzene sulfonic acid reveals changes in the peristaltic movements and architecture of the gut [163]. As in human and rodent IBD, zebrafish IBD is associated with over-expression of proinflammatory cytokines (IL-1β, IL10, and TNF-α), infiltration of the gut by lymphocytes and granulocytes, and microbial dysbiosis [165,166]. A zebrafish gut with IBD-like colitis contains an increased proportion of Proteobacteria and relatively few Firmicutes [167]. This microbial dysbiosis affects the composition of infiltrating leukocytes and is necessary for the induction of enterocolitis, a phenotype observed in human and rodent IBD as well [168].

Over the years, much emphasis has been laid on microbiota manipulation in metabolic diseases. To understand the role of the microbiota in the development of diabetes, Zang et al. developed diet-induced diabetes type 2 (DT2) models by overfeeding zebrafish for 4 weeks. It has been observed that despite offering the same diet, overfeeding in DT2 group induced hyperglycemia and insulin resistance, a phenotype also associated with a decline in the gut microbiota richness in the DT2 zebrafish. Furthermore, several amino acid metabolic pathways (arginine, phenylalanine, and proline) were also downregulated in the DT2 zebrafish, depicting taxonomic and functional similarities in the gut microbiota of DT2 zebrafish and humans [169].

Similarly, Semova and colleagues [141] elucidated the role of the microbiota in intestinal absorption and metabolism of nutrients via in vivo imaging of fluorescent-labeled fatty acids in GF and conventional obese zebrafish. The gut microbiota, dominated by Firmicutes, was shown to enhance fatty acid absorption, along with the accumulation of fat droplets in the lacteal and hepatic portal circulations.

## 5. Mice

The house mouse, *Mus musculus*, has long served as a key model for investigating human biology and the pathology of infection [170]. The advantage of using mice over other mammals is tailored to their small body size, large litters, short generation time, and ease of breeding and maintenance. Moreover, genetic analyses have revealed that a variety of their physiological and biochemical processes are similar to those in humans. The ability to create transgenic and knockout mice strains has contributed enormously to our understanding of the roles of specific gene products in both physiological and pathological processes [171].

### 5.1. The Murine Gut and Microbiota

Murine models provide excellent tools for studying human diseases associated with the gut microbiota. The anatomy of the murine gastrointestinal (GI) tract is similar to that of humans, apart from the differences that do exist between both. For instance, the cecum of mice occupies a relatively larger part of the total GI tract, and plays essential roles in plant fermentation and the production of vitamins B and K. Furthermore, the intestinal villi of mice are longer than those in humans, increasing the surface area for nutrient absorption and compensating for the absence of mucosal folds in the small intestine. In addition, the murine colon is smooth, with no division (Figure 1G,H) [172].

Although the mouse and human gut microbiota are both dominated by the Bacteroidetes and Firmicutes phyla [173], they differ at the species level, where many bacterial species that colonize the murine gut are absent in humans [129]. Indeed, Ley and co-workers [174] reported that the gut microbiota of the mouse can differ by as much as 85% from that of humans at the genus level. Moreover, by comparing the murine and human caecal and stool microbiota, Krych et al. [175] concluded that the microbiota profiles of all of mice strains studied so far are unique and share relatively few features with that of humans. At the same time, both the mouse and human microbiota are dominated by the same phyla and share a substantial number of genera, primarily *Lactobacillus*, *Alistipes*, *Turicibacter*, *Clostridium*, *Bacteroides*, and *Blautia* [176,177,178,179,180]. More recently, and based on three sets of published data [181,182,183], Wang and colleagues [184] characterized the presence of 37 core bacterial genera in 101 healthy male and female mice of different ages and strains and found that in 50% of these animals the microbiota is dominated by *Anaerostipes*, *Parabacteroides*, and *Anaerotruncus* (Figure 1I). Thus, even though the core gut microbiota of mice and humans share several features and are yet dissimilar in many ways [185], mice remain the most commonly used mammal for studies on the gut microbiota [173].

### 5.2. Generation of GF Mice

The use of GF mice has been essential for studying host–microbe interactions [186]. Briefly, pups are removed aseptically by caesarean section of the pregnant dam, transferred to a GF foster mother in an isolator while still in the uterine sac, and maintained and bred in the isolator. Thus, the mother of the first generation of mice is not axenic and viruses, bacteria, and microbial metabolites can be transmitted trans-placentally from the mother to the fetus. Further generations of pups are delivered and cleaned inside the isolator to be used for experimental work (Figure 2C). The GF status of mice is monitored regularly using a combination of serology, culturing, and molecular techniques for the detection of viruses, bacteria, and fungi [129,187,188]. An alternative approach couldinvolve the repeated use of broad-spectrum antibiotics to deplete the gut bacteria. In addition, pregnant dams can be also treated with antibiotics in order to minimize the possibility of microbial transfer to the fetus. However, this approach does not eliminate viruses, fungi, or antibiotic-resistant bacterial species. Furthermore, while GF mice generated by the procedure described above exhibit general impairment of their immune system and entero-neuroendocrine development, such animals created by antibiotic treatment maintain several neuroendocrine and metabolic functions and developmental pathways [189,190].

To determine the contribution of gut bacteria to disease development, gnotobiotic mice can be colonized with one or two specific bacteria or a conventional mixed microbiota [129]. For example, colonization with *Bacteroides fragilis* have shown that this bacterium plays a role in immune homeostasis by maintaining a balance between the populations of CD4^+^ T helper (Th1) and Th2 lymphocytes, as well as by directing FoxP3^+^ T regulatory (T reg) cells to the intestine [191,192,193]. In addition, GF mice can be colonized successively with different bacterial strains over the course of several generations, with an observed response to this varying condition [129]. For example, Atarashi et al. [12] inoculated a cocktail of spore-forming *Clostridia* strains orally into GF mice to determine their potential role in the development and activation of T reg cells. Such inoculation during the early stages of life prevented the development of colitis in adult mice, indicating that the microbiota may play a role in averting intestinal inflammation and autoimmune reactions.

In this context, humanized microbiota mice (HMM) are generated by inoculating GF mice with human gut bacteria or feces [194]. Transplanting the microbiota of obese donors or women during the third trimester of their pregnancy into GF mice induces some of the host’s characteristics in the recipient mice, including weight gain, elevated adiposity, and resistance to insulin [195,196]. Similarly, when Smith et al. [197] performed a study wherein a HMM was created by inoculation from a Malawian twin cohort suffering from severe malnutrition, mice initially lost a considerable amount of weight, a phenotype that was recovered through dietary interventions. This suggests that the microbiota may also play an essential role in energy homeostasis.

### 5.3. The Role of the Gut Microbiota in Mice–Pathogen Interactions

As in zebrafish, MAMPs are recognized by the host innate immune system via pattern recognition receptors (PRRs) including TLRs and NLRs. Petnicki-Ocwieja and co-workers [198] reported that Nod2 is expressed at a significantly reduced level in the intestine of GF mice and that normal expression could be restored by microbial recolonization. Furthermore, the intestine of Nod2-deficient mice was more susceptible to colonization by pathogenic microbes. NLRs are activated by the intestinal microbiota and contributes to formation of the inflammasome and to NF- κB signaling. Induction of the inflammasome leads to the activation of caspase-1 and, subsequently, to the maturation of proinflammatory cytokines IL-1β and IL-18 [199]. The gut microbiota of mice in which the NLRP6 inflammasome had been knocked out exhibit an overabundance of *Prevotellaceae*, which enhanced the severity of chemically induced colitis [200]. Likewise, and similar to what has been reported in zebrafish, TLR/MyD88 signaling in mice is essential for the activation of innate immune responses and for the expression of proinflammatory cytokines. Inoculation of MyD88^-/-^GF mice with segmented filamentous bacteria increases the numbers of intestinal IgA-producing plasma cells and Th17 cells [201].

### 5.4. The Role of the Mice Gut Microbiota in Gut-Related Disorders

Recent scientific insights have highlighted potential associations between the gut microbiota and various disease conditions, such as metabolic [202], cardiovascular, and inflammatory disorders [203], as well as cancer [204]. Among the several murine models of IBD, animals that have been genetically modified and/or had colitis induction chemically exhibit alterations in their gut microbiota. Such alterations have been shown to resemble those observed in humans with IBD, with a reduction in diversity and significant shifts in *Enterobacteriaceae* (*Escherichia*), *Bacteroidaceae* (*Bacteroides*), and *Ruminococcaceae* profiles [205,206,207]. A20 is an inhibitor of NF-κB and apoptotic signaling and has been associated with increased susceptibility to IBD in humans [208,209]. In a mouse model susceptible to spontaneous colitis, deletion of A20 in intestinal epithelial and myeloid cells (A20^IEC/myel-KO^) stimulated apoptosis in the epithelium, loss of Paneth and goblet cells, change in the composition of the microbiota, and reduction in bacterial diversity. Interestingly these changes have been shown to be driven by the commensal *Mucispirillum schaedleri*, a bacteria known to colonize the mucus layer of the GI tract of mice [210]. Hudcovic et al. [211] demonstrated that immunodeficient (SCID) GF mice do not develop colitis in response to treatment with dextran sodium sulfate (DSS), whereas corresponding immunocompetent animals do. Similarly, GF IL-10 deficient mice fail to develop colitis, unlike IL-2 deficient GF mice that exhibit spontaneous colitis when colonized with *E. coli*, but not *Bacteroides vulgatus* or both bacterial species together [212]. Additionally, IL-10-deficient mice exposed to *Helicobacter hepaticus*, *rodentium*, or *typhlonious* develop severe colitis [213].

The ability of the microbiota of obese mice to extract energy from the diet is greater than that of non-obese animals. Turnbaugh and colleagues found that, in comparison to lean mice, the microbiota of leptin-knockout (*ob/ob)* animals produced more monosaccharides and short-chain fatty acids that could provide the host with extra energy from otherwise indigestible foods [195]. Mice fed a high-fat diet have a reduced ratio of Bacteroides:Firmicutes in their gut microbiota, as well as a larger population of Proteobacteria [214]. Furthermore, transplantation of the microbiota from mice in which obesity had been induced by dietary means into lean GF mice increases the adiposity of the recipients to a greater extent than transplantation from lean mice.

GF mice have also been employed in attempts to understand potential correlations between the development of tumors and gut microbiota. Indeed, the microbiota was shown to reduce the severity of intestinal tumors in GF mice as compared to conventional ones [215]. Moreover, knocking out genes encoding p53 and the β chain of the T-cell receptor (TCR) in GF mice exhibit an elevated frequency of spontaneous colorectal tumors [216]. Current investigations are ongoing to examine the potential role of the intestinal microbiota in the development of adenocarcinomas in the ileocecum and cecum of β chain of TCR and p53 deficient mice. Arthur and colleagues [217] examined the role of *E. coli* in inducing invasive carcinoma in GF IL10-deficient mice, with or without a polyketide synthase genotoxic island, and reported reductions in the number and invasiveness of tumors, in combination with microbial dysbiosis involving expansion of microorganisms with genotoxic potential. Such observations demonstrate clearly that the gut microbiota plays a role both in priming the immune system and in carcinogenesis.

## 6. Conclusions

Drawing parallels between vertebrate and invertebrate model systems has served as a foundation for better understanding of conserved pathways utilized by both microbe and host during health and disease. A complex symbiotic relationship exists between hosts and gut microbes, which—if altered—can lead to immune activation, exacerbation of pathogenic virulence, and promotion of disease progression. Although different in power and downside, the overlap in the microbial communities, host defense factors, and host–microbiota interactions across animal models is remarkable. The advantage of *Drosophila*’s simplicity in genetics and microbial diversity presents it as a feasibly manipulable host that could be readily altered in a laboratory setting. Slow development of immune system makes zebrafish an ideal organism for infection pathology and host immune response study. Mice have much genetic and metagenomic resemblance with humans and are by far the most valuable models for analyzing functional host–microbiota relationships. The utilization of these animal models to generate a GF internal environment has paved the way to uncover the role of the microbiota in host–pathogen interactions and in the development of gut-related disorders, an apprehension that could open up for the development of plausible therapeutic approaches for gut dysbiosis-associated diseases.
